# Joint Vehicle Selection and Resource Allocation for Hierarchical Federated Learning in Internet of Vehicles

**DOI:** 10.3390/s26196278

**Published:** 2026-10-03

**Authors:** Zhen Yang, Lihang Wen, Weijing Qi, Lei Guo

**Affiliations:** 1 School of Communications and Information Engineering, Chongqing University of Posts and Telecommunications, Chongqing 400065, China; d200101025@stu.cqupt.edu.cn (Z.Y.); d220101020@stu.cqupt.edu.cn (L.W.); 2 School of Computer Science and Engineering, Northeastern University, Shenyang 110819, China; guolei@cse.neu.edu.cn

**Keywords:** hierarchical federated learning, Internet of Vehicles, resource allocation, vehicle selection

## Abstract

Hierarchical federated learning (HFL) in the Internet of Vehicles (IoV) faces challenges in participation reliability and aggregation efficiency due to vehicle mobility and resource heterogeneity. This paper investigates joint vehicle selection and resource allocation in a three-tier HFL architecture. In this framework, roadside units (RSUs) perform synchronous edge aggregation, while a macro base station (MBS) conducts asynchronous global aggregation. We derive a participation probability based on mobility, local computing capability, and statistical channel state information to construct the eligible vehicle set before final vehicle selection and channel allocation. Furthermore, we design an age-of-model (AoM)-aware policy to mitigate the impact of stale edge models. To maximize HFL training efficiency, we model the system as a multi-agent Markov decision process and propose a multi-agent discrete soft actor–critic (MA-DSAC) algorithm. Simulations on CIFAR-10 and Fashion-MNIST verify that MA-DSAC improves delay-constrained convergence, reducing the delay required to reach the target accuracy by up to 70% and maintaining a lower average AoM than the considered baselines.

## 1. Introduction

Rapid advances in the Internet of Vehicles (IoV), mobile edge computing, and artificial intelligence have enabled various intelligent transportation applications [1]. Connected vehicles continuously generate heterogeneous data through onboard sensors, cameras, and communication devices. Such data support autonomous driving, traffic prediction, and environmental perception. Conventional centralized learning requires vehicles to upload raw data to remote servers, which creates considerable communication overhead and core-network congestion, as well as privacy risks regarding vehicle trajectories and driving behaviors [2,3,4]. Federated learning (FL) addresses these issues by allowing vehicles to train local models without sharing raw data, thereby avoiding raw-data transmission and protecting sensitive information [5]. FL therefore provides a privacy-preserving learning foundation for intelligent vehicular services.

However, conventional FL still requires frequent communication between numerous vehicles and a remote parameter server, which inevitably increases wireless-link loads as the network expands. To improve scalability, hierarchical federated learning (HFL) has been introduced by placing roadside units (RSUs) between vehicles and the central server. Despite its architectural advantages, the direct deployment of HFL in practical IoV networks faces two critical challenges. First, at the edge level, vehicle mobility causes rapid changes in positions and channel conditions. Because RSU coverage is limited, a vehicle may depart before completing local training and model upload [6,7]. Such unsuccessful participation wastes both computing and radio resources. Second, at the global level, heterogeneous computing capabilities and channel conditions lead to unequal edge-model update frequencies across RSUs [8,9]. To prevent fast RSUs from waiting for slower ones, the macro base station (MBS) typically employs asynchronous aggregation (i.e., updating the global model immediately upon receiving an edge model). While this asynchronous mechanism can reduce synchronization delays, it inevitably introduces model staleness, which impairs aggregation efficiency and reliable convergence. Moreover, conventional FL requires frequent communication between numerous vehicles and a remote parameter server. Long-distance model transmission increases wireless-link loads and core-network congestion as the network expands. HFL improves scalability by placing RSUs between vehicles and the central server. Minimizing per-round delay alone cannot represent the learning benefits produced by different vehicle-selection decisions.

Existing FL and HFL studies have examined asynchronous coordination [10], multilevel resource management [11], client selection and clustering [12,13], and resource allocation in air–ground networks [14]. Other work has addressed communication-efficient training [15], as well as joint resource allocation, aggregation, and scheduling in vehicular networks [16,17,18,19,20]. Although asynchronous aggregation reduces synchronization delays among heterogeneous participants, delayed updates may become stale and impair model convergence. Existing remedies include arrival-based partial aggregation [21], adaptive gradient aggregation with bias correction [22], and age-of-information-aware gradient weighting with resource allocation [23]. However, such methods mainly measure freshness at the client-update or gradient level. In the considered HFL architecture, the MBS asynchronously receives edge models produced by different RSUs rather than individual client updates. The freshness of each edge model depends on the time elapsed since the corresponding RSU last completed a successful aggregation. Moreover, heterogeneous RSU update frequencies amplify freshness disparities across edge models. Consequently, client-level age cannot fully characterize RSU-level model staleness. The resulting mismatch motivates an edge-model freshness mechanism designed for asynchronous HFL.

Nevertheless, these existing methods exhibit two major limitations when applied to the considered dynamic vehicular environments. On the one hand, regarding reliable participation, existing methods do not jointly characterize vehicle residence time, local training delay, uplink delay, and statistical channel state information (CSI). Consequently, successful participation in RSU-side synchronous aggregation remains insufficiently modeled. On the other hand, regarding model freshness, current staleness-aware schemes cannot be directly applied to the RSU-level edge models. In the considered HFL architecture, the MBS receives edge models generated asynchronously by different RSUs. The freshness of an edge model depends on its most recent successful RSU-side aggregation, and heterogeneous RSU update frequencies further exacerbate freshness differences. Therefore, client-level update age cannot fully characterize staleness in the RSU-level edge model.

Therefore, this paper investigates joint vehicle selection and channel allocation for HFL in dynamic IoV environments. The considered system consists of one MBS, multiple RSUs equipped with edge servers, and a set of mobile vehicles. Each vehicle performs local training within the coverage area of its associated RSU and uploads the resulting local model to that RSU. Each RSU synchronously aggregates the successfully received local models, whereas the MBS asynchronously aggregates the edge models uploaded by different RSUs. Vehicle arrivals are modeled as a Poisson process, while vehicle speeds follow a truncated Gaussian distribution. By jointly considering the remaining residence time, local computing capability, and statistical CSI, we derive the probability that a vehicle can complete local training and model uploading before leaving the RSU coverage area and use this probability to guide vehicle selection. To quantify edge-model freshness, we introduce the age of model (AoM) and design an AoM-aware asynchronous aggregation policy at the MBS. We then formulate a joint optimization problem to maximize HFL training efficiency, defined as the ratio of the accumulated global-loss reduction to the accumulated training delay. To address the coupling among binary decision variables and the time-varying vehicular environment, the problem is modeled as a multi-agent Markov decision process, and a multi-agent discrete soft actor–critic (MA-DSAC) algorithm is developed for its solution. The main contributions of this paper are summarized as follows:

- We develop a mobility-aware three-tier HFL framework consisting of an MBS, multiple RSUs, and mobile vehicles, where synchronous edge aggregation is performed at the RSUs and asynchronous global aggregation is performed at the MBS. By jointly considering the remaining residence time, local training delay, and uplink transmission delay under statistical CSI, we derive the probability that a vehicle can successfully participate in synchronous edge aggregation. This probability is used as an eligibility-screening criterion before the MA-DSAC policy performs final vehicle selection and channel allocation.
- We introduce AoM to characterize the freshness of RSU-level edge models and design an AoM-aware asynchronous aggregation policy to mitigate the adverse effect of unequal edge-model update rates across RSUs. Based on this mechanism, we formulate a joint vehicle selection and channel allocation problem that maximizes HFL training efficiency, defined as the ratio of the accumulated global-loss reduction to the accumulated training delay.
- We formulate the resulting dynamic discrete optimization problem as a multi-agent Markov decision process and develop an MA-DSAC algorithm in which each RSU jointly determines vehicle selection and channel allocation according to its local observations. The proposed algorithm incorporates discrete stochastic policies, clipped double-Q learning, entropy regularization, and adaptive temperature adjustment to improve exploration and training stability. Experiments on Fashion-MNIST and CIFAR-10 evaluate the proposed scheme in terms of training efficiency, system delay, and model convergence.

The remainder of this paper is organized as follows. Section 2 introduces the vehicle mobility model, delay model, and HFL model; Section 3 formulates the joint vehicle selection and resource allocation optimization problem; Section 4 presents the proposed MA-DSAC algorithm; Section 5 provides simulation results; and Section 6 concludes the paper.

## 2. System Model

**Figure 1:**
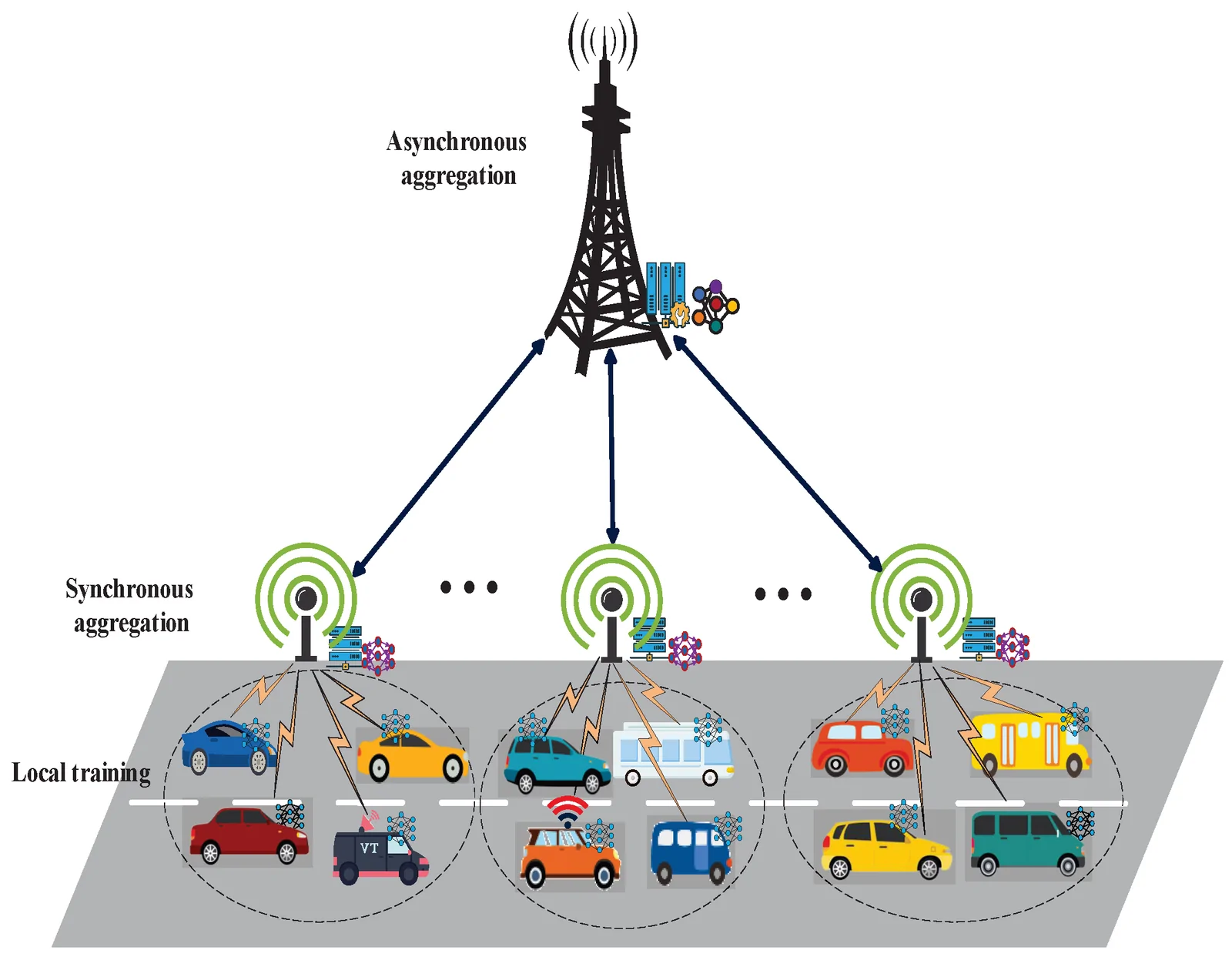
Framework of hierarchical federated learning.

We consider an HFL system deployed in a vehicular network, which consists of an MBS, *K* RSUs denoted by $R=\left\{R_{k}|R_{1},R_{2},\ldots ,R_{K}\right\}$, and *N* vehicles represented by $U=\left\{U_{n}|U_{1},U_{2},\ldots ,U_{N}\right\}$. As illustrated in Figure 1, the vehicles move along one road, with the RSUs on the roadside covered by the MBS. The RSUs are responsible for supporting vehicle-to-RSU (V2R) communications, and each RSU is connected to the MBS via an optical fiber link.

### 2.1. Vehicle Mobility Model

We assume that vehicles periodically upload their speed and location information. Their speeds are independent and identically distributed and are denoted by $V=\left\{V_{n}|n=1,2,\ldots ,N\right\}$. The speeds follow a truncated Gaussian distribution over $\left[V_{\min},V_{\max}\right]$, where $V_{\min}$ and $V_{\max}$ are the minimum and maximum vehicle speeds, respectively. Vehicle arrivals follow a Poisson process with an average arrival rate of λ. Each vehicle maintains a constant speed, and all vehicles move in the same direction.

The speed ($V_{n}$) of vehicle $U_{n}$ follows a truncated Gaussian distribution. According to [24], the probability density function is obtained by normalizing the standard normal distribution on the interval of $\left[V_{\min},V_{\max}\right]$ as shown below:

(1) $$f\left(V_{n}\right)=\left\{\begin{array}{cc} \frac{e^{-\frac{1}{2\delta^{2}}{\left(V_{n}-\mu \right)}^{2}}}{\sqrt{2\pi \delta^{2}}\left(erf\left(\frac{V_{\max}-\mu }{\delta \sqrt{2}}\right)-erf\left(\frac{V_{\min}-\mu }{\delta \sqrt{2}}\right)\right)} & ,V_{\min}⩽V_{n}⩽V_{\max},\\ 0 & ,otherwise. \end{array}\right.$$

where $\delta^{2}$ is the variance, μ is the mean, and $erf\left(\frac{V_{n}-\mu }{\delta \sqrt{2}}\right)$ is the Gaussian error function. By combining the truncated Gaussian distribution with the Poisson process, the dynamic environment in a vehicular network can be simulated more accurately.

### 2.2. Delay Model

Local computing and model aggregation affect the model update delay, while communication delay mainly arises from model transmission between the vehicles and the RSUs over wireless networks. Therefore, to fully evaluate the performance of the HFL system, both computing and transmission delays during the training process should be considered.

#### 2.2.1. Delay in Local Training

Serving as edge nodes, each RSU receives the global model from the MBS. Then, the global model is transmitted to the selected vehicles covered by RSU $R_{k}$ for local training. Let $\alpha_{n,k}\left(i\right)$ denote the vehicle selection indicator, where $\alpha_{n,k}\left(i\right)=1$ means that vehicle $U_{n}$ participates in local training and $\alpha_{n,k}\left(i\right)=0$ otherwise. To evaluate the pre-selection feasibility of a candidate vehicle without introducing a circular dependence on $\alpha_{n,k}\left(i\right)$, the potential local-training delay of vehicle $U_{n}$ at the *i*-th training round is expressed as

(2) $$T_{n,k}^{loc}\left(i\right)=E\frac{c_{n}\left|D_{n,k}\right|}{f_{n}},$$

where *i* indexes an RSU-side scheduling/training round and *g* indexes an asynchronous MBS aggregation event. One round may contain no global update or several global updates. Let $g_{i}$ count the MBS events completed at the beginning of round *i*, and let $v_{k}\left(i\right)\leq g_{i}$ identify the global-model version available to RSU $R_{k}$ when it starts that round. Its selected vehicles initialize their local models from $\omega (v_{k}\left(i\right))$. Here, $c_{n}$ is the number of central processing unit (CPU) cycles required by vehicle $U_{n}$ during local training on one data sample, $|D_{n,k}|$ is the number of data samples at vehicle $U_{n}$, $f_{n}$ is the CPU frequency of vehicle $U_{n}$, and *E* is the number of iterations per round of local training.

#### 2.2.2. Delay in Model Transmission

Since the RSUs and the MBS are interconnected via optical fiber links, the delay in uploading edge models from the RSUs to the MBS and in distributing global models from the MBS to the RSUs is negligible. Consequently, the delay in model transmission of the HFL system primarily consists of two parts: the delay for local model uploading and the delay for global model downloading between the vehicles and the RSUs.

We assume that the communications between the vehicles and the RSUs in the HFL system employ orthogonal frequency-division multiple access (OFDMA) technology. The channel allocation set is denoted by $\rho_{k}\left(i\right)=\left\{\rho_{n,k,m}\left(i\right)|\rho_{n,k,1}\left(i\right),\ldots ,\rho_{n,k,M}\left(i\right)\right\}$, where $\rho_{n,k,m}\left(i\right)$ is the channel allocation indicator. If the *m*-th channel of RSU $R_{k}$ is allocated to vehicle $U_{n}$, $\rho_{n,k,m}\left(i\right)=1$; otherwise, $\rho_{n,k,m}\left(i\right)=0$.

The communications between vehicle $U_{n}$ and RSU $R_{k}$ involve uploading local models and receiving global models. Let $U_{k}\left(i\right)\subseteq U$ denote the set of vehicles covered by RSU $R_{k}$ in round *i*. To avoid ambiguity between the fading amplitude, channel power gain, fading-distribution parameter, and receiver noise power, we denote the small-scale fading amplitude as $a_{n,k}\left(i\right)$ and the composite uplink channel power gain as

(3) $$G_{n,k}\left(i\right)=\beta_{n,k}\left(i\right){\left|a_{n,k}\left(i\right)\right|}^{2},$$

where $\beta_{n,k}\left(i\right)=g_{k}{\left(\frac{d_{0}}{dis\left(R_{k},U_{n}\right)}\right)}^{\ell }$ is the large-scale path gain, $g_{k}$ is the path-loss constant, *ℓ* is the path-loss exponent, $d_{0}$ is the reference distance, and $dis\left(R_{k},U_{n}\right)$ is the distance between RSU $R_{k}$ and vehicle $U_{n}$ [25]. The amplitude ($a_{n,k}\left(i\right)$) follows a Rayleigh distribution with scale parameter $\sigma_{h}$. Thus, ${\left|a_{n,k}\left(i\right)\right|}^{2}$ follows an exponential distribution, and $G_{n,k}\left(i\right)$ follows an exponential distribution with a mean of $\Omega_{n,k}\left(i\right)=2\sigma_{h}^{2}\beta_{n,k}\left(i\right)$. The receiver noise power is denoted by $\sigma_{z}^{2}$. The uplink rate from vehicle $U_{n}$ to RSU $R_{k}$ is given by

(4) $$r_{n,k}^{U}\left(i\right)=W\sum_{m=1}^{M}\rho_{n,k,m}\left(i\right)\log_{2}\left(1+\frac{p_{n,k}G_{n,k}\left(i\right)}{\sigma_{z}^{2}}\right),$$

where *W* is the bandwidth of each uplink channel and $p_{n,k}$ is the transmission power of vehicle $U_{n}$. The upload delay of the local models for vehicle $U_{n}$ is given by

(5) $$T_{n,k}^{U}\left(i\right)=\frac{|d_{n,k}^{loc}\left(i\right)|}{r_{n,k}^{U}\left(i\right)},$$

where $|d_{n,k}^{loc}\left(i\right)|$ is the size of the local model.

Each RSU broadcasts the same global model once to the selected vehicles within its coverage area over a preconfigured downlink broadcast channel. The downlink bandwidth and transmission power are fixed and are therefore not included in the resource-allocation variables considered in this work. Let ${\bar{r}}_{k}^{D}$ denote the fixed effective broadcast rate of RSU $R_{k}$. Accordingly, the RSU-level downlink rate is

(6) $$r_{k}^{D}\left(i\right)={\bar{r}}_{k}^{D}.$$

The corresponding common broadcast delay is defined at the RSU level as

(7) $$T_{k}^{D}\left(i\right)=\frac{|d^{glo}\left(i\right)|}{{\bar{r}}_{k}^{D}},$$

where $|d^{glo}\left(i\right)|$ is the size of the global model. The common delay has no vehicle index because one broadcast serves all selected vehicles, and it is counted only once in each active edge-training round.

#### 2.2.3. Delay in Model Aggregation

In the proposed HFL system, model aggregation consists of two stages: edge aggregation and global aggregation. Edge aggregation is performed by the RSUs, while global aggregation is performed by the MBS. Due to the high computing power of the servers at the MBS and the RSUs, the delays associated with edge model aggregation and global model aggregation are neglected.

Let $S_{k}\left(i\right)=\left\{U_{n}\in U_{k}\left(i\right):\alpha_{n,k}\left(i\right)=1\right\}$ denote the selected vehicles. A nonempty selection receives one broadcast, trains locally, and uploads under the allocation fixed before training. The round reserves the maximum selected-vehicle training-plus-upload duration. A departing vehicle whose upload is incomplete contributes no model. An empty selection initiates neither a broadcast nor a training operation. Thus,

(8) $$T_{k}\left(i\right)=\left\{\begin{array}{cc} T_{k}^{D}\left(i\right)+\max_{U_{n}\in S_{k}\left(i\right)}\left[T_{n,k}^{\mathrm{loc}}\left(i\right)+T_{n,k}^{U}\left(i\right)\right], & S_{k}\left(i\right)\neq ⌀,\\ 0, & S_{k}\left(i\right)=⌀. \end{array}\right.$$

Let $t_{i}$ denote modeled system time at the beginning of scheduling round *i*, with $t_{1}=0$. The admitted RSUs start their round in parallel, so $T_{\mathrm{sys}}\left(i\right)=\max_{k}T_{k}\left(i\right)$, rather than $\sum_{k}T_{k}\left(i\right)$. An RSU that finishes early can upload immediately but waits until $t_{i+1}$ to start its next scheduling decision. Thus, scheduling is round-based, whereas MBS aggregation is arrival-driven within the round. The MBS does not wait for all admitted RSUs before accepting an available edge model. If every RSU is idle, the scheduler advances by $T_{\mathrm{idle}}=0.1$ s without a model update. In either case, $t_{i+1}=t_{i}+T_{\mathrm{sys}}\left(i\right)$. This clock accounts for modeled computation and communication durations, not the host computer’s execution time.

The general availability condition is $v_{k}\left(i\right)\leq g_{i}$. Under the negligible MBS–RSU distribution delay assumed here, Algorithm 1 uses the latest version at a scheduling boundary—namely, $v_{k}\left(i\right)=g_{i}$. This base version remains fixed during the local training operation, even if newer global versions become available before its upload completes.

### 2.3. HFL Model

The proposed HFL framework consists of four steps: vehicle selection, local training, edge aggregation, and global aggregation. In the first step, the vehicles are selected for HFL training based on their movements and channel conditions. It is noted that the edge aggregation is performed synchronously, while the global aggregation is performed asynchronously. Finally, each RSU broadcasts the updated global model once to its selected vehicles for the next round of local training.
**Algorithm 1:** MA-DSAC-Based Joint Vehicle Selection and Resource Allocation for HFL
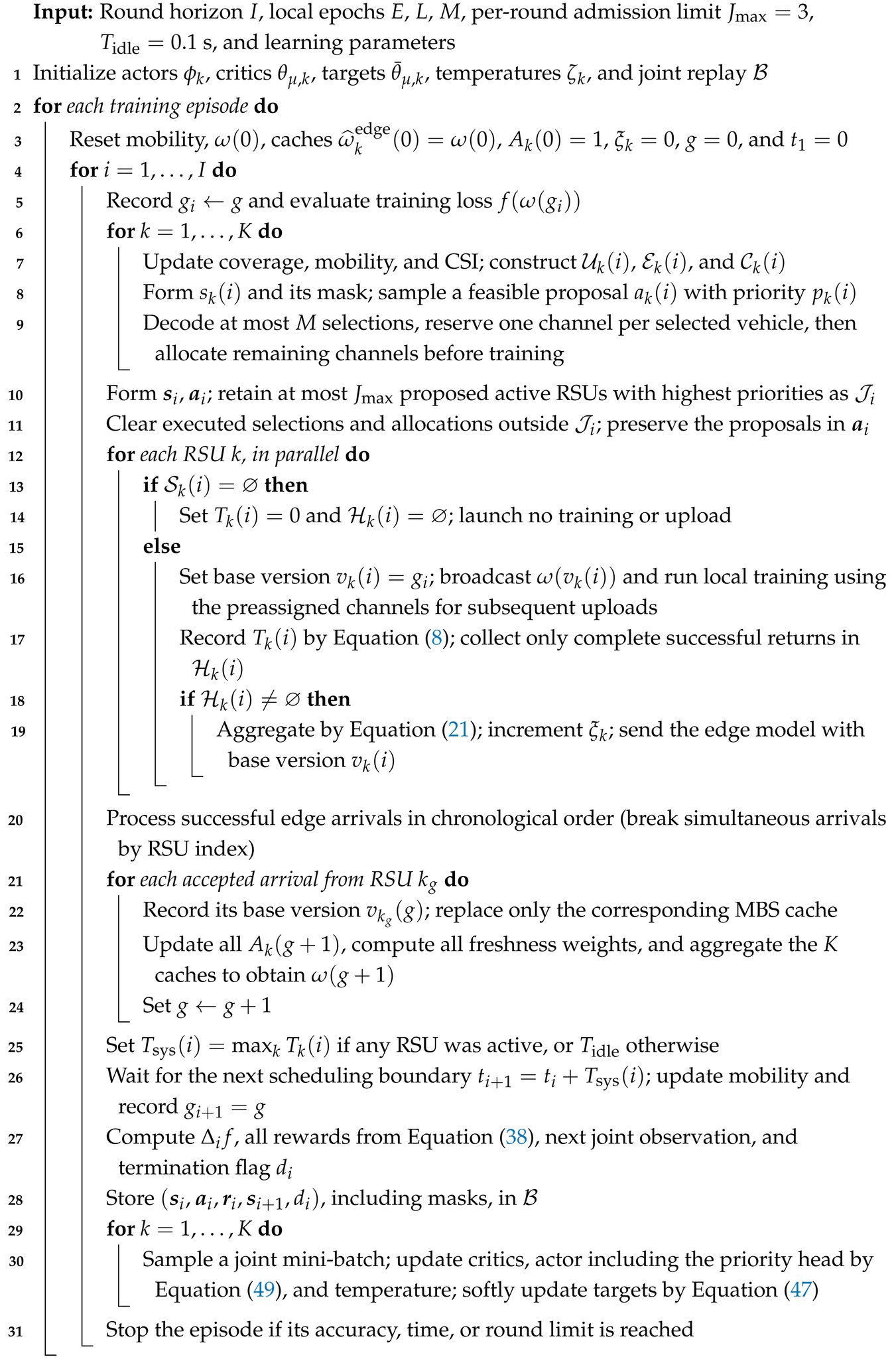


#### 2.3.1. Vehicle Selection

Due to the mobility of the vehicles, the information about their locations and channel conditions is constantly changing. As a result, the vehicles may leave the coverage of the RSU before completing the HFL training, leading to inefficient training processes and resource wastage. Consequently, it is crucial to take into account the duration that each vehicle remains within the coverage area of its associated RSU. Assume that the coverage diameter of RSU $R_{k}$ is D and its position coordinate is $O\left(0,0\right)$. The current position of vehicle $U_{n}$ is $B_{n}\left(X_{n,a}\left(i\right),Y_{n,a}\left(i\right)\right)$, and its position when leaving RSU $R_{k}$ is $C_{n}\left(X_{n,b}\left(i\right),Y_{n,a}\left(i\right)\right)$. Therefore, the minimum stay time of vehicle $U_{n}$ within the coverage area of RSU $R_{k}$ is expressed as

(9) $$T_{n,k}^{stay}\left(i\right)=\frac{|X_{n,b}\left(i\right)-X_{n,a}\left(i\right)|}{V_{n}}.$$

In the HFL system, vehicle $U_{n}$ can participate in synchronous aggregation only if its remaining stay time covers the downlink broadcast, local computing, and model uploading. After vehicle selection and channel allocation are determined, the maximum duration for local computing and model uploading, excluding the common broadcast already counted in Equation (8), is defined as follows

(10) $$T_{k}^{\mathrm{sum}}\left(i\right)=\left\{\begin{array}{cc} \max_{U_{n}\in S_{k}\left(i\right)}\left(T_{n,k}^{\mathrm{loc}}\left(i\right)+T_{n,k}^{U}\left(i\right)\right), & S_{k}\left(i\right)\neq ⌀,\\ 0, & S_{k}\left(i\right)=⌀. \end{array}\right.$$

The quantity of $T_{k}^{\mathrm{sum}}\left(i\right)$ is used to characterize the realized edge-aggregation delay after scheduling. In contrast, the pre-selection successful participation probability must be computed before the final vehicle-selection variable ($\alpha_{n,k}\left(i\right)$) and channel-allocation variable ($\rho_{n,k,m}\left(i\right)$) are determined.

We define the successful participation probability of vehicle $U_{n}$ associated with RSU $R_{k}$ as $\varrho_{n,k}\left(i\right)$. At the beginning of round *i*, RSU $R_{k}$ evaluates this probability using the residence time, local computing delay, model size, transmit power, and statistical CSI. The residence time in Equation (9) is measured before the downlink broadcast. Hence, the time available for uplink transmission after receiving the model and completing local training is

(11) $$\Delta T_{n,k}\left(i\right)=T_{n,k}^{stay}\left(i\right)-T_{k}^{D}\left(i\right)-T_{n,k}^{loc}\left(i\right).$$

This subtraction counts the common downlink delay once and makes the success condition consistent with Equation (8). Because the final channel allocation is not yet known during pre-selection, a conservative one-channel transmission rate is used for reliability screening:

(12) $${\bar{r}}_{n,k}^{U}\left(i\right)=W\log_{2}\left(1+\frac{p_{n,k}G_{n,k}\left(i\right)}{\sigma_{z}^{2}}\right).$$

Then, the successful participation probability is given by

(13) $$\varrho_{n,k}\left(i\right)=P\left(T_{k}^{D}\left(i\right)+T_{n,k}^{loc}\left(i\right)+\frac{|d_{n,k}^{loc}\left(i\right)|}{{\bar{r}}_{n,k}^{U}\left(i\right)}⩽T_{n,k}^{stay}\left(i\right)\right).$$

When $\Delta T_{n,k}\left(i\right)>0$, the minimum channel power gain required for successful uploading is

(14) $$G_{n,k}^{th}\left(i\right)=\frac{\sigma_{z}^{2}}{p_{n,k}}\left(2^{\frac{|d_{n,k}^{loc}\left(i\right)|}{W\Delta T_{n,k}\left(i\right)}}-1\right).$$

Since $G_{n,k}\left(i\right)$ follows an exponential distribution with a mean of $\Omega_{n,k}\left(i\right)$, the successful participation probability is reformulated as

(15) $$\varrho_{n,k}\left(i\right)=\left\{\begin{array}{cc} 0, & \Delta T_{n,k}\left(i\right)⩽0,\\ \exp\left(-\frac{G_{n,k}^{th}\left(i\right)}{\Omega_{n,k}\left(i\right)}\right), & \Delta T_{n,k}\left(i\right)>0. \end{array}\right.$$

Given the reliability threshold (φ), the eligible vehicle set at RSU $R_{k}$ is defined as

(16) $$E_{k}\left(i\right)=\left\{U_{n}\in U_{k}\left(i\right)|\varrho_{n,k}\left(i\right)⩾\varphi \right\}.$$

Thus, $\varrho_{n,k}\left(i\right)$ is used only for eligibility screening. The final vehicle selection and channel allocation are still determined by the MA-DSAC policy. The decision order is statistical channel evaluation, eligibility screening, vehicle selection, and channel allocation, and followed local training and model uploading. Selection and allocation are decoded from the same policy action before training; channel allocation is not a second decision made after training.

#### 2.3.2. Learning Process in HFL

It has been proven that stale models can negatively impact the convergence of the global model [26]. To mitigate this issue and accelerate convergence, we propose an AoM-based update policy, which is defined as follows.

We measure AoM by global aggregation events rather than wall-clock time. The global model changes only when the MBS performs an aggregation. Thus, the number of intervening aggregation events directly describes how far an RSU-level edge model may lag behind the current global learning trajectory. An elapsed interval without a global update increases system delay but does not increase the global-model version difference. The physical-time cost is separately captured by the local-training, transmission, and accumulated-delay terms. This definition distinguishes cache freshness from elapsed training time. AoM is observed at scheduling epochs but updated only at accepted MBS aggregation events.

Let *g* denote the index of an asynchronous global aggregation event. The MBS maintains one cached edge model, denoted by ${\hat{\omega }}_{k}^{\mathrm{edge}}\left(g\right)$, for each RSU ($R_{k}$). At event *g*, the MBS receives an edge model from RSU $R_{k_{g}}$ and replaces the corresponding cached model. The AoM of each RSU is then updated as

(17) $$A_{k}\left(g+1\right)=\left\{\begin{array}{cc} 1, & k=k_{g},\\ A_{k}\left(g\right)+1, & k\neq k_{g}. \end{array}\right.$$

We adopt a one-based convention. Hence, $A_{k}\left(g\right)=1$ represents the freshest RSU-level edge model, whereas $A_{k}\left(g\right)-1$ is the number of intervening global aggregation events since the latest accepted update from RSU $R_{k}$.

AoM and model staleness describe different aspects of freshness. Let $v_{k_{g}}\left(g\right)$ denote the global-model version used to initialize the edge update that arrives from RSU $R_{k_{g}}$ at event *g*. Its staleness is

(18) $$\eta_{k_{g}}\left(g\right)=g-v_{k_{g}}\left(g\right).$$

AoM characterizes how recently each RSU refreshed its cached edge model. In contrast, staleness measures the version delay of a particular arriving model. These quantities are related but are not generally identical, and $\eta_{k_{g}}\left(g\right)$ is not used as an additional aggregation weight.

After updating all AoM values, the MBS calculates one normalized freshness weight for each cached RSU model as

(19) $${℧}_{k}\left(g+1\right)=\frac{\exp[-A_{k}\left(g+1\right)]}{\sum_{j=1}^{K}\exp\left[-A_{j}\left(g+1\right)\right]},\;k=1,\ldots ,K.$$

The arriving RSU has $A_{k_{g}}\left(g+1\right)=1$, whereas the AoM values of non-arriving RSUs increase. Consequently, the rule preserves the newly refreshed cached model and progressively reduces the weights of cached models that have not been refreshed for several global aggregation events. It does not use the pre-arrival AoM of the newly received model to penalize that model.

Vehicle $U_{n}$ participating in the HFL training downloads the global model from its associated RSU ($R_{k}$), and subsequently performs local training. The objective of the local training process is to minimize the loss function on the set of training samples. We define the loss function for each sample (*j*) as $f_{n}\left(x_{j},y_{j},\omega_{n}\left(\tau \right)\right)$, which is $f_{j}\left(\omega_{n}\left(\tau \right)\right)$ in short. $\omega_{n}\left(\tau \right)$ is the local model, with τ being the number of local model updates, while $x_{j}$ is the input feature vector and $y_{j}$ is the corresponding output label. For each dataset ($D_{n,k}$), at vehicle $U_{n}$, the loss function is given by

(20) $$F_{n}\left(\omega_{n}\left(\tau \right)\right)=\frac{1}{|D_{n,k}|}\sum_{j\in D_{n,k}}f_{j}\left(\omega_{n}\left(\tau \right)\right).$$

Let $H_{k}\left(i\right)\subseteq S_{k}\left(i\right)$ contain exactly the selected vehicles whose local training and complete model upload succeed before they leave the RSU coverage area, including the initial broadcast in the elapsed duration: $T_{k}^{D}\left(i\right)+T_{n,k}^{\mathrm{loc}}\left(i\right)+T_{n,k}^{U}\left(i\right)\leq T_{n,k}^{\mathrm{stay}}\left(i\right)$. This realized success set is distinct from both the eligible set and the selected set. With $\xi_{k}\left(i\right)$ denoting the number of completed edge aggregations before round *i*, the edge update is

(21) $$\omega_{k}\left(\xi_{k}\left(i\right)+1\right)=\frac{\sum_{U_{n}\in H_{k}\left(i\right)}\left|D_{n,k}\right|\;\omega_{n}^{\mathrm{loc}}\left(i\right)}{\sum_{U_{n}\in H_{k}\left(i\right)}\left|D_{n,k}\right|},\;H_{k}\left(i\right)\neq ⌀,$$

where $\omega_{n}^{\mathrm{loc}}\left(i\right)$ is trained from $\omega (v_{k}\left(i\right))$. If $H_{k}\left(i\right)=⌀$, no edge aggregation is performed, $\xi_{k}$ is unchanged, and no edge upload is generated. A nonempty selected set alone is insufficient to trigger aggregation.

Before local training, upload admission retains, at most, three active RSUs according to the policy-produced priorities, as specified in Section 4.2 and Algorithm 1. Each retained RSU asynchronously uploads an edge model only when its successful set is nonempty. To reduce the influence of cached edge models that have not been refreshed recently, we propose an AoM-based aggregation policy. After replacing ${\hat{\omega }}_{k_{g}}^{\mathrm{edge}}\left(g\right)$ with the newly received model, the MBS performs global aggregation as follows:

(22) $$\omega \left(g+1\right)=\sum_{k=1}^{K}{℧}_{k}\left(g+1\right){\hat{\omega }}_{k}^{\mathrm{edge}}\left(g+1\right),$$

where ${\hat{\omega }}_{k_{g}}^{\mathrm{edge}}\left(g+1\right)$ is the newly received edge model and ${\hat{\omega }}_{k}^{\mathrm{edge}}\left(g+1\right)={\hat{\omega }}_{k}^{\mathrm{edge}}\left(g\right)$ for $k\neq k_{g}$. Hence, all cached RSU models participate in the aggregation, while models from RSUs that have not uploaded recently receive progressively smaller AoM-based weights. The arriving model was initialized from version $v_{k_{g}}\left(g\right)=g-\eta_{k_{g}}\left(g\right)$ [27]; this version index is retained to describe model staleness but does not introduce an additional aggregation coefficient.

At event *g*, the MBS first updates the cache entry of the source RSU and records the base global-model version of the arriving edge update. It then updates all AoM values, calculates the *K* freshness weights, and aggregates the cached RSU models. The new global model is subsequently broadcast for the next training process. This procedure is repeated until the target model accuracy or training-time limit is reached.

## 3. Problem Formulation

To evaluate global training efficiency, we measure global-loss reduction [28] over a common system-time horizon. We define $\Delta_{i}f=f\left(\omega \left(g_{i}\right)\right)-f\left(\omega \left(g_{i+1}\right)\right)$, where $g_{i+1}-g_{i}$ counts accepted MBS aggregation events during round *i*. If no update is accepted, $g_{i+1}=g_{i}$ and $\Delta_{i}f=0$. The elapsed system time is $t_{I+1}-t_{1}=\sum_{i=1}^{I}T_{\mathrm{sys}}\left(i\right)$, with $T_{\mathrm{sys}}\left(i\right)$ defined in Section 2.2.3. The objective is the accumulated global-loss reduction per unit system training time:

(23) $$P_{1}:\max_{\alpha ,\rho }\frac{\sum_{i=1}^{I}\Delta_{i}f}{\sum_{i=1}^{I}T_{\mathrm{sys}}\left(i\right)}$$

(24) $$s.t.\;\alpha_{n,k}\left(i\right)\in \left\{0,1\right\},\forall n,k,$$

(25) $$\rho_{n,k,m}\left(i\right)\in \left\{0,1\right\},\forall n,k,m,$$

(26) $$\sum_{k=1}^{K}\alpha_{n,k}\left(i\right)⩽1,\forall n,$$

(27) $$\sum_{U_{n}\in U_{k}\left(i\right)}\sum_{m=1}^{M}\rho_{n,k,m}\left(i\right)⩽M,\forall k,$$

(28) $$\sum_{U_{n}\in U_{k}\left(i\right)}\rho_{n,k,m}\left(i\right)⩽1,\forall k,m,$$

(29) $$\alpha_{n,k}\left(i\right)⩽\sum_{m=1}^{M}\rho_{n,k,m}\left(i\right)⩽M\alpha_{n,k}\left(i\right),\forall n,k,$$

(30) $$0<f_{n}⩽f_{n}^{\max},\forall n,$$

(31) $$\alpha_{n,k}\left(i\right)⩽1\left\{\varrho_{n,k}\left(i\right)⩾\varphi \right\},\forall n,k.$$

where *I* is the number of RSU-side scheduling rounds in the considered horizon, not the number of global aggregation events. The arrays ($\alpha =\{\alpha_{n,k}\left(i\right)\}$ and $\rho =\{\rho_{n,k,m}\left(i\right)\}$) collect decisions for $U_{n}\in U_{k}\left(i\right)$, k=1,…,K, m=1,…,M, and i=1,…,I. Decisions outside the associated candidate set are zero. Constraint (26) ensures that each vehicle can associate with at most one RSU. Constraint (27) limits the number of channels for each RSU, and constraint (28) ensures that each channel can be occupied by at most one vehicle. Constraint (29) couples vehicle selection and channel allocation: an unselected vehicle cannot occupy any channel, while a selected vehicle is assigned at least one and at most *M* channels. Constraint (30) represents the computing capability of vehicle $U_{n}$. Constraint (31) ensures that only vehicles whose conservative successful participation probability satisfies the reliability threshold can be selected. This constraint removes the circular dependence between participation-probability evaluation and the final scheduling variables. Problem $P_{1}$ is non-convex and hard to solve directly. Moreover, the strong coupling of the variables makes the situation more challenging. Therefore, the following algorithmic design is intended to obtain an effective approximate scheduling policy for this long-term objective, rather than to provide an exact equivalent transformation of $P_{1}$.

The feasible executed decisions in problem $P_{1}$ additionally satisfy $\sum_{k=1}^{K}1\{|S_{k}\left(i\right)\left|>0\right\}\leq J_{\max}=3$ in every scheduling round. Here, $S_{k}\left(i\right)$ denotes the selected-vehicle set after RSU admission, rather than the proposed set before admission. This restriction is enforced by the priority-based admission rule in Section 4.2 and Algorithm 1.

## 4. Joint Vehicle Selection and Resource Allocation

### 4.1. Problem Transformation

In this section, we adopt a physically motivated two-stage approximation for problem $P_{1}$, rather than claiming a strictly equivalent reformulation. The original problem jointly couples vehicle selection and channel allocation over the long-term HFL training horizon. Directly optimizing the accumulated fractional objective is difficult because the binary scheduling variables, wireless transmission delay, vehicle mobility, and global loss evolution are coupled across rounds. To make the problem tractable for online decision-making, we decompose each round into two sequential decisions that follow the practical HFL procedure: vehicle selection is decoded first, and channel allocation is decoded conditionally on that selection, both before local training. The allocation is then used for the subsequent local-model uploads.

Based on this decision order, the approximate decomposition contains an outer vehicle-selection subproblem ($P_{outer}$) and an inner channel-allocation subproblem ($P_{inner}$). The outer subproblem selects reliable participants to improve the loss-delay tradeoff, while the inner subproblem allocates uplink channels to reduce the transmission delay of the selected vehicles. The two subproblems remain coupled through the selected vehicle set and the realized training delay, and the proposed MA-DSAC algorithm learns the corresponding online scheduling policy from the interaction with the HFL environment.

(32) $$\begin{array}{cc} P_{outer}: & \max_{\alpha }\frac{\Delta_{i}f}{T_{k}^{D}\left(i\right)+\max_{U_{n}\in S_{k}\left(i\right)}\left(E\frac{c_{n}\left|D_{n,k}\right|}{f_{n}}+\frac{|d_{n,k}^{loc}\left(i\right)|}{b_{n,k}\log_{2}\left(1+\frac{p_{n,k}G_{n,k}\left(i\right)}{\sigma_{z}^{2}}\right)}\right)}\\ s.t.\; & (\mathrm{24}),(\mathrm{26}),(\mathrm{30}),(\mathrm{31}). \end{array}$$

(33) $$\begin{array}{cc} P_{inner}: & \min_{\rho }\max_{U_{n}\in S_{k}\left(i\right)}\left(\begin{array}{c} E\frac{c_{n}\left|D_{n,k}\right|}{f_{n}}\\ \;+\frac{|d_{n,k}^{loc}\left(i\right)|}{W\sum_{m=1}^{M}\rho_{n,k,m}\left(i\right)\log_{2}\left(1+\frac{p_{n,k}G_{n,k}\left(i\right)}{\sigma_{z}^{2}}\right)} \end{array}\right)\\ s.t.\; & (\mathrm{25}),(\mathrm{27}),(\mathrm{28}),(\mathrm{29}). \end{array}$$

(1) Outer Vehicle Selection Subproblem: Before local training begins, each RSU selects appropriate participants from candidate vehicles for the current round of the HFL. Vehicle selection accounts for the vehicles’ mobility and computing capabilities and restricts the candidates to those whose estimated completion probabilities satisfy the prescribed reliability threshold. This probabilistic screening does not guarantee successful completion for every selected vehicle; only successful returns enter edge aggregation. In this subproblem formulation, the communication-related parameters are inherited from the system model. In Equation (32), $b_{n,k}=W$ is a fixed one-channel reference bandwidth consistent with Equation (12), not the final allocated bandwidth. Equation (33) subsequently uses $W\sum_{m}\rho_{n,k,m}\left(i\right)$. Equation (32) is a local loss-delay surrogate, not an exact decomposition of the system-time objective. Its displayed ratio applies to nonempty selections; an empty selection has zero surrogate value. Loss reduction is an outcome learned through interaction, not a known future loss at decision time.

(2) Inner Channel Allocation Subproblem: After completing the local training, the vehicles under each RSU transmit their local models to the corresponding RSU. Channels have already been assigned at the scheduling stage, conditional on the selection, and are used for transmission after training. The channel allocation subproblem is formulated as Equation (33). The common broadcast delay ($T_{k}^{D}\left(i\right)$) is included once in $P_{outer}$. Because it is fixed and independent of ρ, it does not affect the optimizer of $P_{inner}$ and is omitted from Equation (33).

The outer problem has a fractional objective, whereas the inner problem is a discrete min–max delay problem. For an empty selection, the inner allocation is all-zero, and its delay is zero. The subproblems are inherently combinatorial and intractable for conventional optimization methods. Moreover, conventional deep reinforcement learning (DRL) methods, such as deep Q-network (DQN) and proximal policy optimization (PPO), often suffer from unstable training and poor exploration when dealing with the discrete action spaces of vehicle selection and channel allocation. To address these challenges, this paper proposes an MA-DSAC algorithm, which extends soft actor–critic (SAC) into a multi-agent DRL approach [29]. Moreover, an adaptive temperature mechanism is designed to automatically balance exploration and exploitation within each agent, thereby enhancing the robustness of policy learning and stabilizing training in dynamic environments.

### 4.2. Markov Decision Process

In vehicular networks, real-time optimization of the vehicle selection and channel allocation is required in the HFL training to cope with rapidly changing system states. Because the scheduler observes the network and mobility variables required for the current decision but not the complete model parameters, local data distributions, or SGD history, these observations are not asserted to form a mathematically sufficient Markov state. Instead, we model the scheduling problem as an observable-state MDP approximation, in which the unobserved learning evolution is absorbed into the stochastic transitions and rewards [30]. Each RSU acts as an agent that observes the states of its associated vehicles and makes decisions to improve the efficiency of the HFL training. Accordingly, the MDP model tuple is defined as

(34) $$G=\left\{S,A,P,R,\gamma \right\},$$

where S and A denote the joint state and action spaces. For each RSU, $C_{k}\left(i\right)\subseteq E_{k}\left(i\right)\subseteq U_{k}\left(i\right)$ is the policy candidate set, containing at most *L* vehicles. Ordered candidate observations and their validity mask form $s_{k}\left(i\right)$; $a_{k}\left(i\right)$ contains selection, allocation, and an upload priority. We define $s_{i}=\left(s_{1}\left(i\right),\ldots ,s_{K}\left(i\right)\right)$, $a_{i}=\left(a_{1}\left(i\right),\ldots ,a_{K}\left(i\right)\right)$, and $r_{i}=\left(r_{1}\left(i\right),\ldots ,r_{K}\left(i\right)\right)$. Kernel P acts on joint states and actions, and γ∈[0,1) is the discount factor. The three components are defined below.

(1) State Space: All the RSUs collect the local observations of the vehicles within their respective coverage areas, which, together, form the network state. During the *i*-th round of the HFL training, the state ($o_{n}\left(i\right)$) of vehicle $U_{n}$ consists of the following components:

- The current position of vehicle $U_{n}$: $B_{n}\left(X_{n,a}\left(i\right),Y_{n,a}\left(i\right)\right)$;
- The composite uplink channel power gain from vehicle $U_{n}$ to RSU $R_{k}$: $\left\{G_{n,k}\left(i\right)\right\}$;
- The computing capability of vehicle $U_{n}$: $f_{n}$;
- The size of the local model of vehicle $U_{n}$: $|d_{n}^{loc}\left(i\right)|$;
- The conservative probability that vehicle $U_{n}$ completes local training and model upload before leaving the RSU coverage area: $\varrho_{n,k}\left(i\right)$;
- The current AoM value of RSU $R_{k}$ at the beginning of scheduling round *i*: $A_{k}\left(g_{i}\right)$.

At the *i*-th round, the observation corresponding to the state of vehicle $U_{n}$ is given by

(35) $$o_{n}\left(i\right)=\left\{B_{n}\left(X_{n,a}\left(i\right),Y_{n,a}\left(i\right)\right),G_{n,k}\left(i\right),f_{n},\left|d_{n}^{loc}\left(i\right)\right|,\varrho_{n,k}\left(i\right),A_{k}\left(g_{i}\right)\right\}.$$

Candidate observations are ordered consistently within a transition and padded to *L* slots. The mask ($m_{k}\left(i\right)$) identifies valid slots; the next-state mask is stored with the next observation. The local policy input is

(36) $$s_{k}\left(i\right)=\left({\mathrm{stack}}_{U_{n}\in C_{k}\left(i\right)}o_{n}\left(i\right),m_{k}\left(i\right)\right).$$

(2) Action Space: The actor proposes vehicle selections (${\tilde{\alpha }}_{n,k}\left(i\right)$) and channel allocations (${\tilde{\rho }}_{n,k,m}\left(i\right)$), together with an upload priority ($p_{k}\left(i\right)$). These proposals are distinguished from the executed indicators ($\alpha_{n,k}\left(i\right)$ and $\rho_{n,k,m}\left(i\right)$) after system-level admission. The local proposal is

(37) $$a_{k}\left(i\right)=\left({\left\{{\tilde{\alpha }}_{n,k}\left(i\right)\right\}}_{U_{n}\in C_{k}\left(i\right)},{\left\{{\tilde{\rho }}_{n,k,m}\left(i\right)\right\}}_{U_{n}\in C_{k}\left(i\right),\;m=1,\ldots ,M},p_{k}\left(i\right)\right).$$

A direct enumeration of the joint vehicle-selection and channel-allocation action is computationally infeasible. If RSU $R_{k}$ observes $N_{k}$ vehicles and *M* uplink channels, the number of possible joint actions can grow on the order of $2^{N_{k}}{\left(N_{k}+1\right)}^{M}$. Therefore, MA-DSAC uses a candidate-local, masked, and factorized action representation. At the beginning of each round, RSU $R_{k}$ first applies the reliability screening set ($E_{k}\left(i\right)$) and retains at most *L* candidates for policy inference. The actor contains *L* Bernoulli vehicle-selection heads, *M* categorical channel-allocation heads with *L* logits each, and one scalar RSU-upload-priority head. Its sigmoid output ($p_{k}\left(i\right)$) lies in [0,1]. Before local training, let ${\tilde{S}}_{k}\left(i\right)=\left\{U_{n}\in C_{k}\left(i\right):{\tilde{\alpha }}_{n,k}\left(i\right)=1\right\}$ and $A_{i}^{\mathrm{RSU}}=\left\{k:{\tilde{S}}_{k}\left(i\right)\neq ⌀\right\}$ denote proposed selections and active RSUs. Retain all if their number is at most three; otherwise, retain the three highest priorities, breaking ties by RSU index. Let $J_{i}$ denote this admission set. The executed indicators are $\alpha_{n,k}\left(i\right)={\tilde{\alpha }}_{n,k}\left(i\right)1_{\left\{k\in J_{i}\right\}}$ and $\rho_{n,k,m}\left(i\right)={\tilde{\rho }}_{n,k,m}\left(i\right)1_{\left\{k\in J_{i}\right\}}$. Non-admitted RSUs therefore do not train in that round. Admission does not guarantee success: only retained RSUs with $H_{k}\left(i\right)\neq ⌀$ generate an edge upload. If no vehicle is selected by an RSU, its priority is ignored by the admission step. If its candidate set is empty, its priority output is masked to zero. Feasibility masks exclude ineligible vehicles and actions that violate the channel-allocation constraints before sampling. Hence, the per-RSU action-output dimension is $D_{a}=L+LM+1=L\left(M+1\right)+1$, where the final one denotes the upload-priority score. This factorization avoids enumerating all feasible action combinations. Excluding the fixed neural-network forward pass, candidate screening and action decoding require $O(N_{k}+LM)$ operations for one RSU, or $O(K\left(N_{k}+LM\right))$ operations for all RSUs.

The three-RSU limit is a per-round admission constraint: at most three RSUs are authorized to train and subsequently upload in a scheduling round. It is not merely a limit on simultaneous transmissions while additional RSUs train in the background. An admitted RSU with no successful return generates no upload, so the number of accepted MBS events can be smaller than three. During selection decoding, once *M* vehicles have been selected, the remaining selection heads are masked inactive; one distinct channel is reserved for each selected vehicle before the remaining channels are assigned. Consequently, $|{\tilde{S}}_{k}\left(i\right)|\leq \min\left\{L,M\right\}$, and each executed selected vehicle has at least one channel.

(3) Reward Function: The reward combines global-loss reduction over the scheduling interval with the RSU-specific duration. The empty-action case is defined explicitly:

(38) $$r_{k}\left(i\right)=\left\{\begin{array}{cc} \frac{\Delta_{i}f}{f\left(\omega \left(g_{i}\right)\right)\;T_{k}\left(i\right)},\; & S_{k}\left(i\right)\neq ⌀,\;f\left(\omega \left(g_{i}\right)\right)>0,\\ 0, & \mathrm{otherwise}. \end{array}\right.$$

If the loss is zero, the learning target has been attained, and the episode terminates. If no edge model is accepted, $\Delta_{i}f=0$. A failed attempt still incurs its reserved round delay but creates no edge update and does not reset its source RSU’s AoM. An RSU with no executed selection has zero local delay and zero reward; the positive system idle interval prevents an undefined system-time objective when all RSUs are idle.

The rewards share a global-loss signal but are not necessarily equal because their denominators are RSU-specific. We therefore use the term RSU-specific reward. Equation (38) is a dense local surrogate of Equation (23), not an exactly equivalent objective. Its normalization expresses relative loss reduction. The scalar system reward used for reporting is $r_{\mathrm{sys}}\left(i\right)=\sum_{k=1}^{K}r_{k}\left(i\right)$. This reporting sum does not replace the individual reward components used to train the RSU-specific critics.

The loss signal is evaluated on the client-side training partitions: the global model is evaluated on the fixed loaders of the N=100 vehicles, with losses weighted by evaluated sample counts. Test accuracy and test loss are reported separately and are not inputs to the reward.

### 4.3. Design of MA-DSAC Scheme

**Figure 2:**
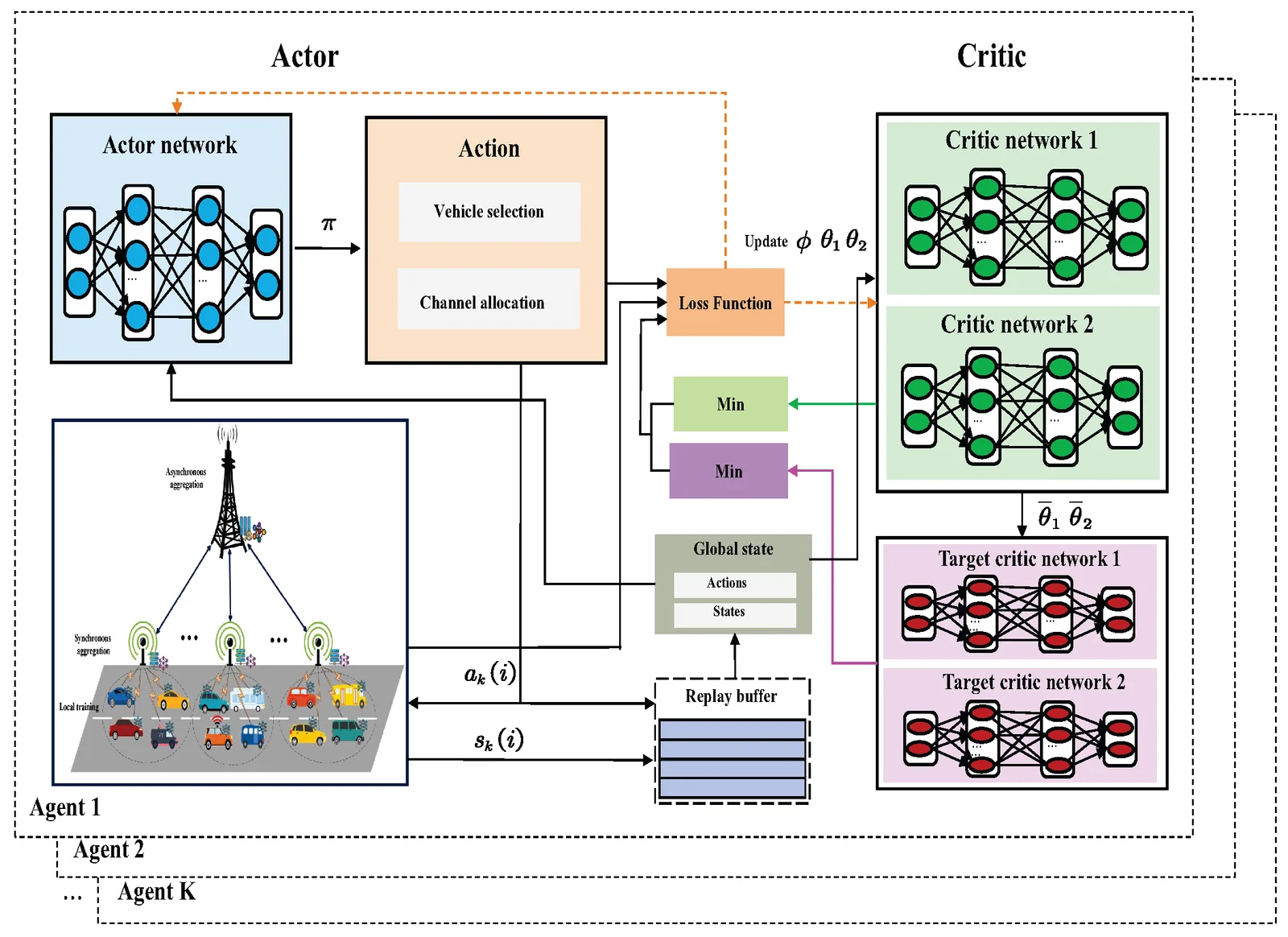
Architecture of the MA-DSAC algorithm.

The architecture of the MA-DSAC algorithm is shown in Figure 2. SAC combines expected return with policy entropy [31]. The local actor outputs discrete scheduling decisions ($u_{k}=\left({\tilde{\alpha }}_{k},{\tilde{\rho }}_{k}\right)$) and an upload-priority score ($p_{k}=p_{\phi_{k}}\left(s_{k}\right)$). The policy probability ($\pi_{\phi_{k}}\left(a_{k}|s_{k}\right)$) and its entropy refer to the discrete scheduling decisions, with the priority score held fixed. For RSU *k*, the policy objective is

(39) $$\pi_{k}^{*}=\arg\max_{\pi_{k}}E_{\pi }\left[\sum_{i=1}^{I}\gamma^{i-1}\left(r_{k}\left(i\right)+\zeta_{k}H\left(\pi_{\phi_{k}}\left(\cdot |s_{k}\left(i\right)\right)\right)\right)\right],$$

where $\zeta_{k}>0$, $\pi =\prod_{j=1}^{K}\pi_{\phi_{j}}$ is the factorized joint policy, and the entropy is evaluated over feasible masked actions. Each RSU has its own actor, two critics, two target critics, and temperature parameters; there is no parameter sharing. During centralized training, the critics use $\left(s_{i},a_{i}\right)$, whereas the actor uses only $s_{k}\left(i\right)$ and its local mask. Cooperation is represented by joint critic inputs and the global-loss component of the RSU-specific rewards, not by identical rewards. Local actor proposals are followed by the system-level upload-priority admission step.

For a fixed joint policy, the RSU-specific soft value functions satisfy

(40) $$Q_{k}\left(s_{i},a_{i}\right)=E\left[r_{k}\left(i\right)+\gamma V_{k}\left(s_{i+1}\right)|s_{i},a_{i}\right],$$

and

(41) $$V_{k}\left(s_{i+1}\right)=E_{a^{\prime }∼\pi }\left[Q_{k}\left(s_{i+1},a^{\prime }\right)-\zeta_{k}\log\pi_{\phi_{k}}\left(a_{k}^{\prime }|s_{k}\left(i+1\right)\right)\right].$$

For a local actor update, we hold the other agents’ policies fixed and define ${\bar{Q}}_{k}^{\mathrm{old}}\left(s,a_{k}\right)=E_{a_{-k}∼\pi_{-k}^{\mathrm{old}}}\left[Q_{k}^{\mathrm{old}}\left(s,\left(a_{k},a_{-k}\right)\right)\right]$. For fixed priority outputs, the discrete-policy projection at a fixed joint state is

(42) $$\pi_{k}^{\mathrm{new}}=\arg\min_{\pi_{k}^{\prime }\in \Pi_{k}}D_{\mathrm{KL}}\left(\pi_{k}^{\prime }\left(\cdot |s_{k}\right)\;∥\;\frac{\exp({\bar{Q}}_{k}^{\mathrm{old}}\left(s,\cdot \right)/\zeta_{k})}{Z_{k}\left(s\right)}\right),$$

where $Z_{k}\left(s\right)$ normalizes the Boltzmann distribution over feasible local actions and $\Pi_{k}$ is the local policy class.

**Lemma** **1** (entropy-regularized policy projection). *For a fixed critic, fixed other-agent policies and priorities, fixed discrete-action support, and $\zeta_{k}>0$, the minimizers in Equation (42) are exactly the maximizers of*

(43) $$E_{a_{k}∼\pi_{k}^{\prime }}\left[{\bar{Q}}_{k}^{\mathrm{old}}\left(s,a_{k}\right)\right]+\zeta_{k}H\left(\pi_{k}^{\prime }\left(\cdot |s_{k}\right)\right).$$

**Proof.**  See Appendix A. This is an equivalence of policy-update objectives. It does not establish monotonic improvement of the learned multi-agent Q-functions under simultaneous updates and function approximation. □

The clipped double-Q formulation [32] uses two RSU-specific centralized critics: $Q_{\theta_{\mu ,k}}\left(s,a\right)$ and μ∈{1,2}. Let $\chi_{i}=\left(s_{i},a_{i},r_{i},s_{i+1},d_{i}\right)$ be a joint replay transition, where $d_{i}=1$ denotes termination and the candidate masks are included in the observations. The joint action records the masked actor proposals; priority admission is a deterministic environmental operation. This preserves the causal mapping from an actor proposal to its reward and next state. The critic objective is

(44) $$J_{Q,k}\left(\theta_{\mu ,k}\right)=E_{\chi_{i}∼B}\left[\frac{1}{2}{\left(Q_{\theta_{\mu ,k}}\left(s_{i},a_{i}\right)-y_{k}\right)}^{2}\right],\;\mu \in \left\{1,2\right\}.$$

The soft target uses freshly sampled next actions ($a_{j}^{\prime }∼\pi_{\phi_{j}}\left(\cdot |s_{j}\left(i+1\right)\right)$) for all RSUs:

(45) $$\begin{array}{cc} y_{k}= & r_{k}\left(i\right)+\gamma \left(1-d_{i}\right)\left[\min_{\mu =1,2}Q_{{\bar{\theta }}_{\mu ,k}}\left(s_{i+1},a^{\prime }\right)-\zeta_{k}\log\pi_{\phi_{k}}\left(a_{k}^{\prime }|s_{k}\left(i+1\right)\right)\right]. \end{array}$$

With the target held constant, the critic gradient is

(46) $$\nabla_{\theta_{\mu ,k}}J_{Q,k}=E_{\chi_{i}∼B}\left[\left(Q_{\theta_{\mu ,k}}\left(s_{i},a_{i}\right)-y_{k}\right)\nabla_{\theta_{\mu ,k}}Q_{\theta_{\mu ,k}}\left(s_{i},a_{i}\right)\right].$$

The target parameters are softly updated:

(47) $${\bar{\theta }}_{\mu ,k}\leftarrow \kappa \theta_{\mu ,k}+\left(1-\kappa \right){\bar{\theta }}_{\mu ,k},\;\mu \in \left\{1,2\right\}.$$

For actor updates, sample joint observations from replay and fresh feasible actions from the current actors. Holding the other actors and critics fixed, minimize

(48) $$J_{\pi ,k}\left(\phi_{k}\right)=E_{s∼B,\;a∼\pi }\left[\zeta_{k}\log\pi_{\phi_{k}}\left(a_{k}|s_{k}\right)-\min_{\mu =1,2}Q_{\theta_{\mu ,k}}\left(s,a\right)\right].$$

Writing $Q_{k}^{\min}=\min_{\mu =1,2}Q_{\theta_{\mu ,k}}\left(s,a\right)$, the actor gradient is

(49) $$\begin{array}{cc} \nabla_{\phi_{k}}J_{\pi ,k}= & E[\nabla_{\phi_{k}}\log\pi_{\phi_{k}}\left(u_{k}|s_{k}\right)\left(\zeta_{k}\log\pi_{\phi_{k}}\left(u_{k}|s_{k}\right)-Q_{k}^{\min}\right)\\ \; & \;-\frac{\partial Q_{k}^{\min}}{\partial p_{k}}\nabla_{\phi_{k}}p_{\phi_{k}}\left(s_{k}\right)]. \end{array}$$

The expectation has the same sampling distribution as Equation (48). The two terms update the discrete scheduling policy and the priority score, respectively. The priority gradient passes through the centralized critic and the sigmoid output, not through the hard top-three admission operation. Empty-candidate priorities are masked to zero and receive no gradient.

The entropy temperature is adapted through

(50) $$J_{\zeta ,k}\left(\zeta_{k}\right)=E_{s∼B,\;a_{k}∼\pi_{\phi_{k}}}\left[-\zeta_{k}\left(\log\pi_{\phi_{k}}\left(a_{k}|s_{k}\right)+\bar{H}\right)\right],$$

where $\bar{H}$ is the target entropy. Its gradient is

(51) $$\nabla_{\zeta_{k}}J_{\zeta ,k}=-E_{s∼B,\;a_{k}∼\pi_{\phi_{k}}}\left[\log\pi_{\phi_{k}}\left(a_{k}|s_{k}\right)+\bar{H}\right].$$

The parameter updates for each RSU are

(52) $$\begin{array}{cc} \theta_{\mu ,k} & \leftarrow \theta_{\mu ,k}-\lambda_{Q}\nabla_{\theta_{\mu ,k}}J_{Q,k},\\ \phi_{k} & \leftarrow \phi_{k}-\lambda_{\phi }\nabla_{\phi_{k}}J_{\pi ,k},\\ \zeta_{k} & \leftarrow \zeta_{k}-\lambda_{\zeta }\nabla_{\zeta_{k}}J_{\zeta ,k}, \end{array}$$

where temperature updates are constrained to $\zeta_{k}>0$. Algorithm 1 separates the scheduling index, accepted MBS events, and joint replay updates.

## 5. Simulation Results

### 5.1. Experiment Setup

Experimental Platform: We consider a scenario comprising an MBS, 100 vehicles, and 6 RSUs, with each RSU covering a circular area with a radius of 500 m. The hardware platform includes an Intel(R) Core(TM) i7-13650HX CPU and an NVIDIA GeForce RTX 4060 graphics processing unit (GPU). The software stack is Python 3.8 and PyTorch 2.4.1+cu121 on 64-bit Windows 10 Professional.

To quantify the online decision delay of MA-DSAC, we repeatedly executed the complete scheduling procedure for all K=6 RSUs on the CPU, using a batch size of one for each RSU. The measured procedure included candidate screening, action masking, actor-network inference, and action decoding. After 300 warm-up runs, 2000 measured runs yielded a mean delay of 2.85 ms and a 95th-percentile delay of 3.58 ms.

Datasets and Models: We consider CIFAR-10 (https://www.cs.toronto.edu/~kriz/cifar.html (accessed on 27 September 2026)) and Fashion-MNIST (https://github.com/Eeshababar/FMINIST-Classification (accessed on 27 September 2026)). (1) CIFAR-10 consists of 60,000 color images, each with a resolution of 32×32, categorized into 10 classes with 6000 images per class (50,000 for training and 10,000 for testing). (2) Fashion-MNIST contains 70,000 grayscale images of fashion products across 10 classes, each with a resolution of 28×28, with 60,000 training images and 10,000 test images.

**Figure 3:**
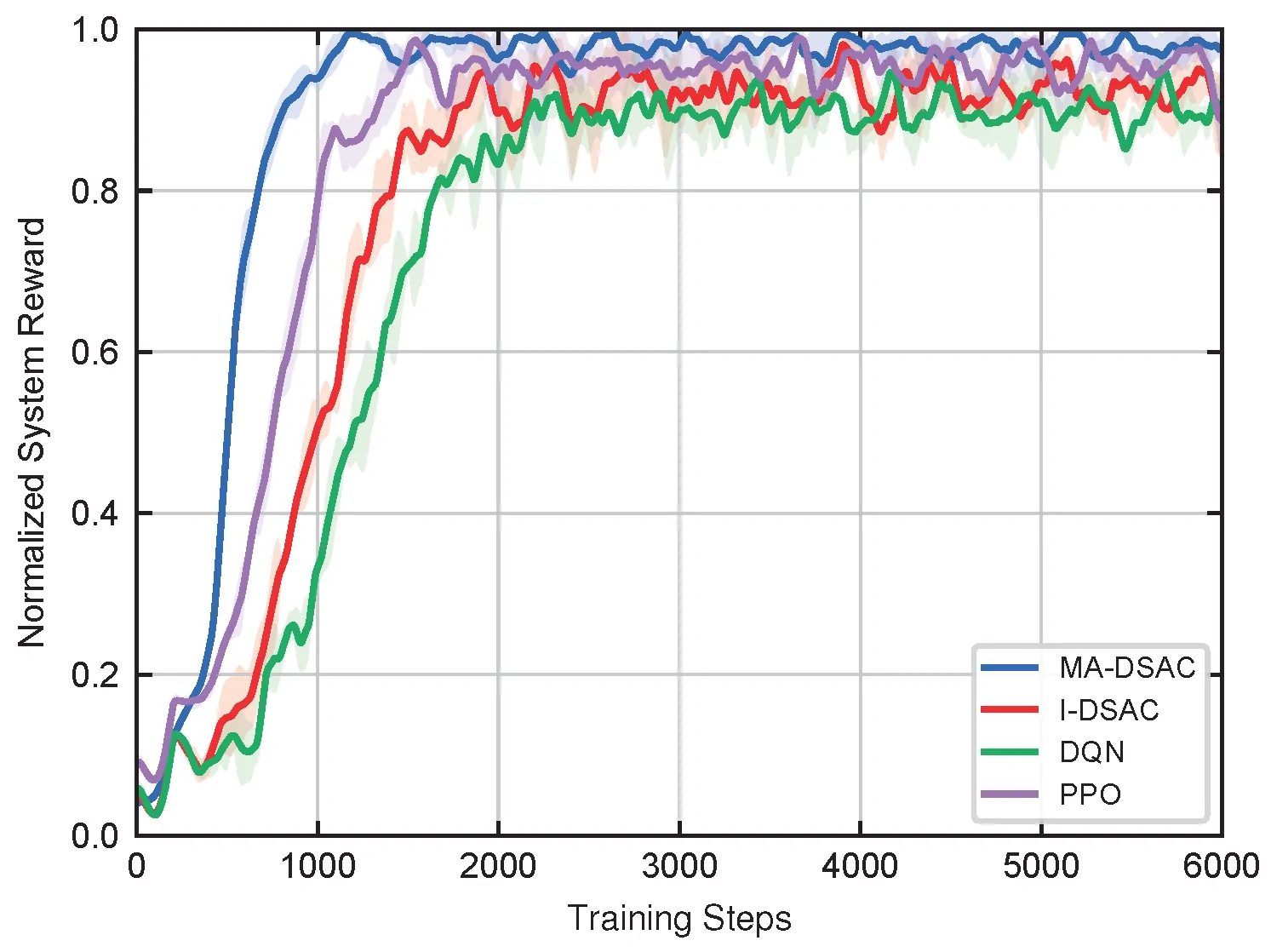
Convergence of the normalized system reward for different DRL algorithms on CIFAR-10 versus environmental steps.

**Figure 4:**
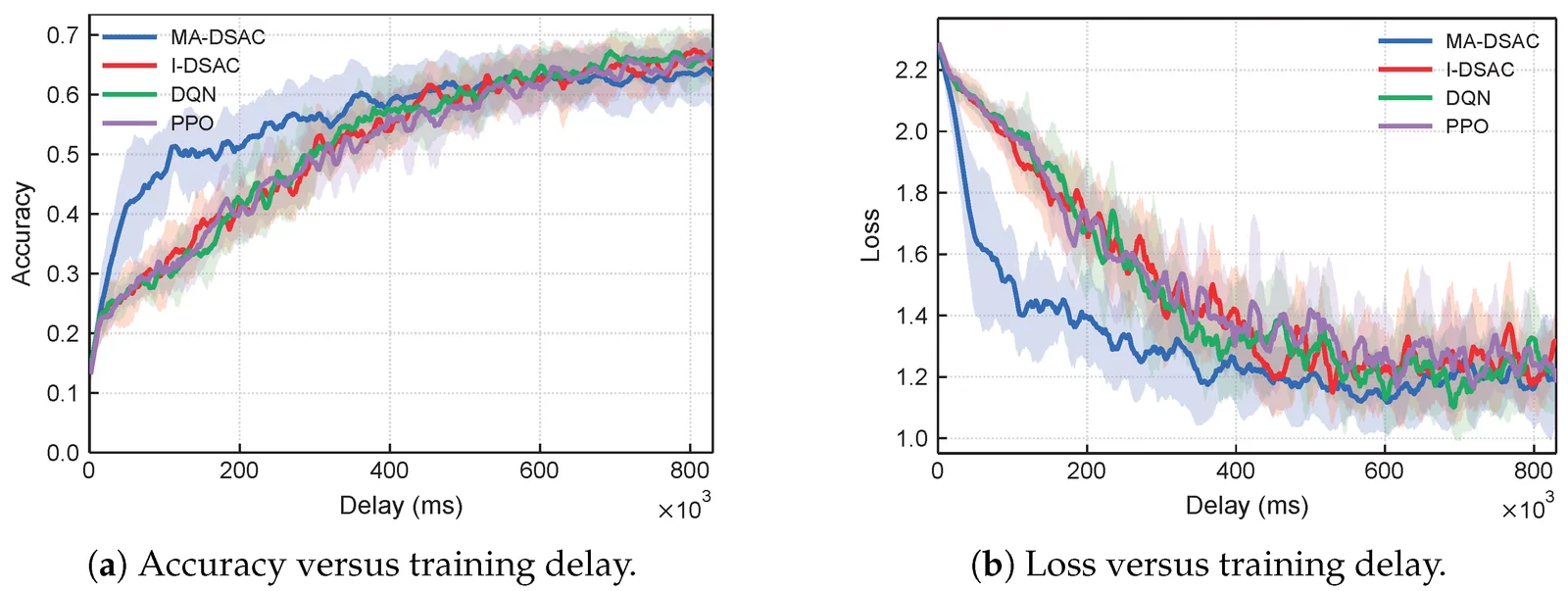
Learning performance of different algorithms on CIFAR-10 versus training delay: (**a**) accuracy and (**b**) loss.

**Figure 5:**
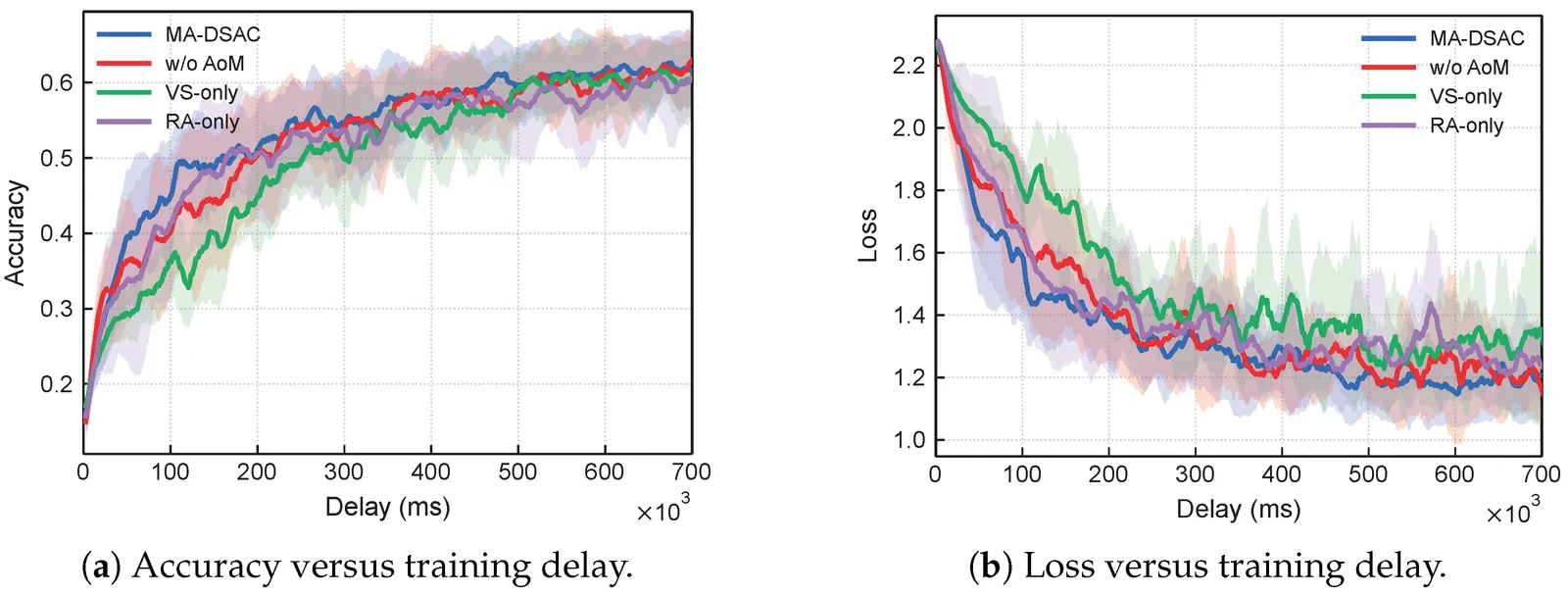
Learning performance of different schemes on CIFAR-10 versus training delay: (**a**) accuracy and (**b**) loss.

**Figure 6:**
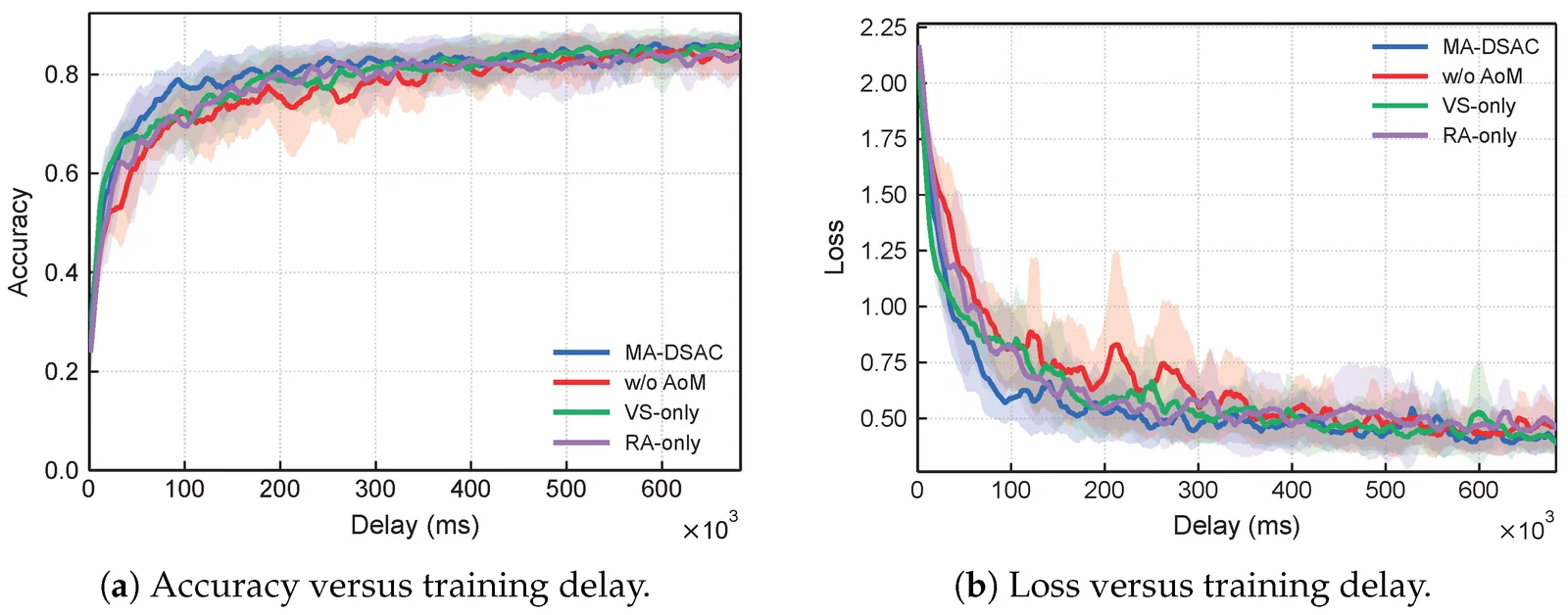
Learning performance of different schemes on Fashion-MNIST under an RSU-level Dirichlet non-IID partition (concentration 0.10) versus training delay: (**a**) accuracy and (**b**) loss.

**Figure 7:**
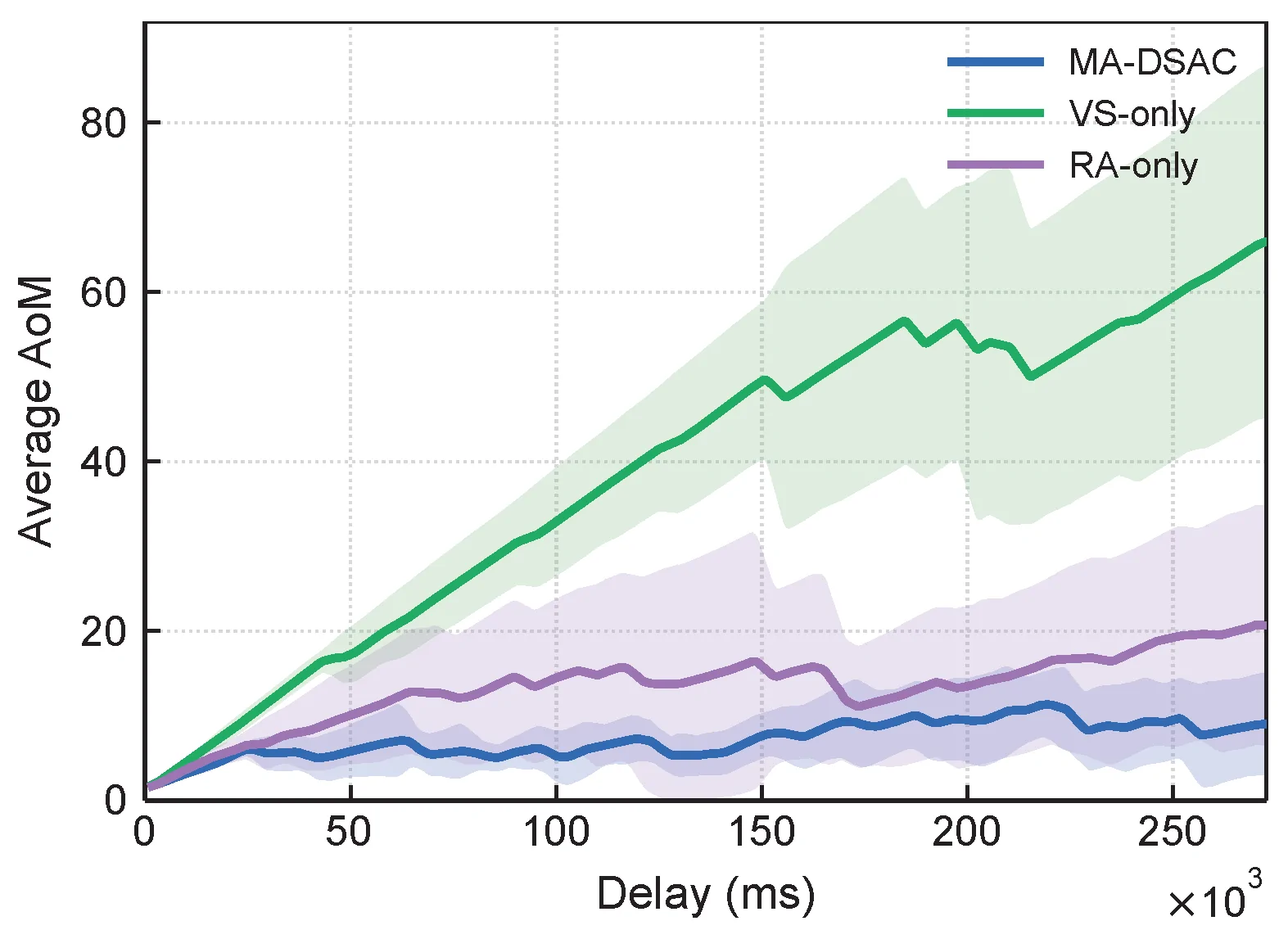
Average AoM of different scheduling configurations on CIFAR-10 versus training delay.

The CIFAR-10 experiments in Figure 3, Figure 4 and Figure 5 use disjoint, equal-sized IID partitions: the training samples are randomly shuffled and uniformly divided among the N=100 vehicles, with 500 samples per vehicle. In contrast, the Fashion-MNIST experiment in Figure 6 uses an RSU-level Dirichlet non-IID label partition with a concentration parameter of 0.10. For each class, its training samples are first distributed among the six RSU data pools according to a Dirichlet draw, then divided approximately evenly among the vehicles initially associated with each RSU. The vehicle-to-dataset assignment is fixed throughout a simulation run; the initial vehicle–RSU association is used only to construct the RSU-level statistical partition and does not cause local data to be reassigned as vehicles subsequently move. This construction introduces statistical heterogeneity across RSUs while preserving the complete training set, with an average of 600 samples per vehicle. The original test sets are retained at the MBS for global-model evaluation. The CIFAR-10 experiment in Figure 7 uses the same IID partition as the experiments in Figure 3, Figure 4 and Figure 5. All results in Figure 3, Figure 4, Figure 5, Figure 6 and Figure 7 are averaged over three independent runs using random seeds 2, 3, and 4; the solid curves represent the across-seed mean, and the shaded regions indicate one standard deviation. Following the parameter-reporting scope of recent wireless FL, HFL, and vehicular FL studies [13,31,33], Table 1 reports the system, mobility, computation, and communication parameters, while Table 2 reports the FL model, data partition, local-training configuration, and MA-DSAC hyperparameters.

To evaluate the performance of the proposed MA-DSAC framework, we compare it with two categories of benchmark schemes: representative DRL algorithms and structural ablation variants.

(1) DRL Benchmark Algorithms:

- I-DSAC: Independent Discrete Soft Actor–Critic scheme. In this baseline, each RSU agent independently learns its vehicle selection and channel allocation policy using a single-agent discrete soft actor–critic (DSAC) algorithm, without cooperative multi-agent state sharing or coordination [31].
- DQN: The Deep Q-Network algorithm is a widely adopted value-based DRL benchmark where each RSU employs a deep neural network with an ϵ-greedy exploration strategy to map vehicular states to discrete joint decision actions [34].
- PPO: The Proximal Policy Optimization algorithm is a state-of-the-art policy-gradient baseline adapted for discrete action spaces, utilizing clipped surrogate objectives to maintain stable policy updates [35].

(2) Structural Ablation Variants:

- VS-only: Vehicle-Selection-only scheme. This variant solely executes mobility-aware vehicle selection based on residence-time probabilities while allocating available channels uniformly among selected vehicles without dynamic channel optimization.
- RA-only: Resource-Allocation-only scheme. This scheme performs dynamic channel allocation to minimize transmission delays but omits residence-time feasibility validation during vehicle selection.
- w/o AoM: Proposed scheme without Age-of-Model weighting. This variant executes asynchronous global aggregation at the MBS using standard dataset-size-proportional weights, ignoring the freshness differences among RSU-level edge models.

To ensure a fair comparison, all benchmark algorithms and variants are evaluated under identical vehicular mobility traces, wireless channel dynamics, and computation/communication resource constraints.

### 5.2. Performance of the MA-DSAC Scheme

For Figure 3, the step-level system reward is the sum of the *K* RSU-specific rewards, i.e., $r_{\mathrm{sys}}\left(i\right)=\sum_{k=1}^{K}r_{k}\left(i\right)$. Each algorithm uses 30 episodes of 200 scheduling rounds, totaling 6000 environmental steps (Table 2). One environmental step is one round; the Training Steps axis counts continuously across episodes. An episode return sums the 200 step-level system rewards within one episode. Figure 3 processes the step-level sequence directly, without first summing rewards within each training episode. Inactive RSUs contribute zero; the reporting sum does not replace $r_{k}\left(i\right)$ in the critic targets.

For visualization, in Figure 3, each algorithm–seed step-level reward trace is first converted to a rolling cumulative reward with a window of 200 consecutive environmental steps and independently min–max-scaled to [0,1] using the minimum and maximum of that trace; the bounded trace is then smoothed for display. The window moves along the concatenated step-level sequence without resetting at episode boundaries; its length of 200 steps does not imply prior aggregation into episode returns. Thus, Figure 3 shows within-method policy-training evolution rather than absolute reward magnitudes across different algorithms. Figure 3 compares the normalized system rewards of MA-DSAC, I-DSAC, DQN, and PPO. All algorithms initially obtain low rewards because the agents must explore a large discrete scheduling space. MA-DSAC reaches a normalized reward of approximately 0.9 after 700 environmental steps. PPO, I-DSAC, and DQN require approximately 1100, 1400, and 1600 environmental steps, respectively, to reach a comparable normalized level within their respective traces. These levels do not imply equal absolute rewards across algorithms because each trace is normalized independently. MA-DSAC subsequently stabilizes near 1.0 and exhibits smaller fluctuations than the benchmark algorithms. Such behavior is consistent with the clipped double-Q structure, entropy regularization, and adaptive temperature adjustment. The combined mechanisms support efficient exploration and limit Q-value overestimation. Therefore, the following performance comparisons use well-converged scheduling policies rather than transient action decisions.

Figure 4 compares the CIFAR-10 learning performance against accumulated training delay. Table 3 quantifies the first-attainment delay at three target accuracies. At targets of 0.40 and 0.50, MA-DSAC reduces the mean delay by 60.9–65.2% and 66.5–70.2%, respectively, relative to I-DSAC, DQN, and PPO. At 0.60, the reduction narrows to 12.4–17.4%, while the standard deviation of MA-DSAC increases. Thus, the primary benefit is faster early and intermediate learning, whereas the methods become closer at higher accuracy. Figure 4b shows the same trend in test loss: MA-DSAC decreases the loss more rapidly at small accumulated delays, and the gap narrows during later training.

Figure 5 and Figure 6 present the ablation results on CIFAR-10 with IID data and Fashion-MNIST with an RSU-level Dirichlet non-IID label distribution, respectively. MA-DSAC achieves faster accuracy growth and test-loss reduction during the early and intermediate training stages in both settings. The performance gaps gradually narrow as training proceeds, indicating comparable late-stage learning performance among all schemes. Therefore, the proposed components mainly improve delay-constrained convergence rather than asymptotic model accuracy. VS-only applies completion-probability-based vehicle selection but cannot adapt channel allocation to heterogeneous channel conditions. RA-only dynamically allocates channels but may serve vehicles with high departure risks because completion-probability screening is absent. The w/o AoM variant retains joint scheduling but assigns no freshness-dependent weights during asynchronous global aggregation. Consequently, stale edge models may exert excessive influence on early global updates. The Fashion-MNIST results show that the complementary benefits of reliable participation, adaptive channel allocation, and freshness-aware aggregation persist under the evaluated non-IID partition. This evidence is limited to the RSU-level Dirichlet setting with concentration 0.10 and does not establish robustness to all forms or degrees of statistical heterogeneity.

Figure 7 evaluates the effects of vehicle selection and channel allocation on RSU-level model freshness. MA-DSAC maintains the average AoM below approximately 12 throughout the displayed delay range. Near the final observation point, VS-only, RA-only, and MA-DSAC produce average AoM values of approximately 65, 20, and 8, respectively. VS-only exhibits an overall increasing trend because uniform channel allocation cannot accommodate time-varying channel conditions and transmission demands. RA-only lowers AoM through adaptive channel allocation, but the absence of departure-risk screening still limits scheduling efficiency. MA-DSAC combines completion-probability-based vehicle selection with adaptive channel allocation, thereby supporting timely uploads and more frequent RSU-side aggregation. AoM weighting does not directly reduce the AoM values shown in Figure 7. Instead, freshness-aware weighting limits the influence of residual stale edge models during asynchronous global aggregation. Therefore, joint scheduling maintains fresher edge models, while AoM-aware aggregation converts model freshness into the convergence improvements observed in Figure 5 and Figure 6.

## 6. Conclusions

This paper studied joint vehicle selection and resource allocation for HFL in dynamic IoV environments. A three-tier MBS–RSU–vehicle architecture supports synchronous edge aggregation at each RSU and asynchronous global aggregation at the MBS. To improve participation reliability, we derived each vehicle’s completion probability based on its remaining residence time, training delay, uplink delay, and statistical CSI. An AoM-aware aggregation rule further limits the influence of stale edge models on global model updates. We formulated the coupled scheduling problem as a multi-agent Markov decision process. MA-DSAC was then developed using clipped double-Q learning, entropy regularization, and adaptive temperature tuning. Experiments on CIFAR-10 with IID data and Fashion-MNIST with an RSU-level Dirichlet non-IID partition demonstrate faster scheduling-policy training and improved delay-constrained learning performance. On CIFAR-10, MA-DSAC reduces the mean delay required to reach 0.50 accuracy by 66.5–70.2% compared with I-DSAC, DQN, and PPO. MA-DSAC also maintains the average AoM below 15 throughout the evaluated delay range. Near the final observation point, MA-DSAC achieves an average AoM of approximately 8. The corresponding values of VS-only and RA-only are approximately 65 and 20, representing reductions of 88% and 60%, respectively. The results show that vehicle selection and adaptive channel allocation jointly improve the timeliness of edge-model updates. AoM-aware aggregation further limits the influence of stale edge models during asynchronous global aggregation. Together, the three components improve HFL training efficiency by coordinating reliable participation, communication resources, and edge-model freshness. These conclusions are limited to the evaluated straight-road, access-link-dominated setting with single-RSU association, reliable and timely mobility reports, and error-free transmissions once the modeled rate and residence-time conditions are satisfied. Heterogeneous RSU–MBS backhaul and edge-server delays, handover and overlapping coverage, retransmissions, and mobility-prediction errors are not modeled. Future work will investigate theoretical convergence and broader statistically heterogeneous settings, including different Dirichlet concentrations, feature skew, and fairness across RSUs, and extend the framework to adaptive scheduling in space–air–ground integrated network (SAGIN)-assisted vehicular networks.

## Figures and Tables

**Table 1:** System, mobility, and communication parameters.

Parameter	Value
Number of MBSs/RSUs/vehicles, 1/K/N	1/6/100
Number of uplink channels, *M*	12
Maximum admitted RSUs per round, $J_{\max}$	3
All-idle scheduling interval, $T_{\mathrm{idle}}$	0.1 s
RSU coverage diameter, D	1000 m
Vehicle speed, $V_{n}$	$N(70,5^{2})$ truncated to [60,80] km/h
Poisson road-entry rate, λ	0.33 vehicle/s
Vehicle CPU frequency, $f_{n}$	[1,2] GHz
CPU cycles per sample, $c_{n}$	$1.0\times 10^{6}$ cycles/sample
Uplink-channel bandwidth, *W*	0.833 MHz
Pre-selection reference bandwidth, $b_{n,k}$	*W* (one channel)
Vehicle uplink/RSU downlink power	23 dBm/30 dBm
Effective RSU broadcast rate, ${\bar{r}}_{k}^{D}$	100 Mbps
Noise power, $\sigma_{z}^{2}$	$3.32\times 10^{-15}$ W
Reference distance, $d_{0}$	1 m
Path-loss exponent, *ℓ*	2.2
Reliability threshold, φ	0.90

**Table 2:** Federated-learning and MA-DSAC implementation settings.

Category	Parameter	Setting
FL model	CIFAR-10 CNN	C(3,32)–MP–C(32,64)–MP–FC(4096,128)–FC(128,10)
FL model	Fashion-MNIST CNN	C(1,32)–MP–C(32,64)–MP–FC(3136,128)–FC(128,10)
FL model	Parameters/model payload	CIFAR-10: 545,098/2.08 MiB; Fashion-MNIST: 421,642/1.61 MiB; FP32
FL training	Data partition	CIFAR-10: IID uniform, 500 samples/vehicle; Fashion-MNIST: RSU-level Dirichlet non-IID, concentration 0.10, 600 samples/vehicle on average; test sets at MBS
FL training	Batch size/local epochs	B=32; E=5
FL training	Optimizer/learning rate/loss	SGD; momentum =0.9; $\eta =10^{-2}$; cross-entropy
FL training	Independent runs/random seeds	Three runs; 2, 3, and 4
MA-DSAC	Actor	Input: L×7+ mask; MLP: 256–256, ReLU; factorized heads
MA-DSAC	Priority-head update	Sigmoid score; gradient through the centralized critic, Equation (49)
MA-DSAC	Twin critics for each RSU *k*	Joint (s,a); MLP: 256–256, ReLU; scalar $Q_{\theta_{\mu ,k}}$
MA-DSAC	Candidate cap/action-output dimension	L=20; $D_{a}=261$
MA-DSAC	Optimizer/mini-batch size	Adam/256
MA-DSAC	Learning rates	$\lambda_{\phi }=10^{-3}$; $\lambda_{Q}=10^{-2}$; $\lambda_{\zeta }=10^{-2}$
MA-DSAC	Discount/buffer/soft update	γ=0.99; $\left|B\right|=5\times 10^{4}$; κ=0.005
MA-DSAC	Initial temperature/target entropy	$\zeta_{0}=0.10$; $\bar{H}=49.50$
MA-DSAC	Warm-up/update frequency	1000 interactions/one update per environment step
DRL training	Training episodes	30
DRL training	Scheduling rounds per episode	I=200; MBS event count *g* is separate
DRL training	Total training environment steps	30×200=6000

C$\left(C_{\mathrm{in}},C_{\mathrm{out}}\right)$ denotes a 3×3 convolutional layer with a stride of 1 and padding of 1, followed by ReLU activation. MP denotes 2×2 max pooling. FC denotes a fully connected layer. Model payloads are calculated using 32-bit floating-point parameters.

**Table 3:** Cumulative delay required to reach multiple CIFAR-10 target accuracies.

Method	0.40	0.50	0.60
MA-DSAC	49.3±26.1	72.8±27.3	306.6±208.7
I-DSAC	125.9±32.1	217.1±60.5	349.8±83.6
DQN	136.3±32.8	217.9±58.8	365.5±90.4
PPO	141.4±43.9	243.8±52.8	371.3±121.0

Values are the mean ± standard deviation of the first-attainment delay over random seeds 2, 3, and 4, in units of $10^{3}\;\mathrm{ms}$.

## Data Availability

The CIFAR-10 and Fashion-MNIST datasets used in this study are publicly available at https://www.cs.toronto.edu/~kriz/cifar.html (accessed on 27 September 2026) and https://github.com/zalandoresearch/fashion-mnist (accessed on 27 September 2026), respectively. The data-partition and implementation settings are described in Section 5.1.

## Abbreviations

The following abbreviations are used in this manuscript:

AoM	Age of Model
CIFAR-10	Canadian Institute for Advanced Research 10-class dataset
CPU	Central Processing Unit
CSI	Channel State Information
DNN	Deep Neural Network
DQN	Deep Q-Network
DRL	Deep Reinforcement Learning
DSAC	Discrete Soft Actor–Critic
FL	Federated Learning
GPU	Graphics Processing Unit
HFL	Hierarchical Federated Learning
I-DSAC	Independent Discrete Soft Actor–Critic
non-IID	Non-Independent and Identically Distributed
IoV	Internet of Vehicles
KL	Kullback–Leibler
MA-DSAC	Multi-Agent Discrete Soft Actor–Critic
MBS	Macro Base Station
MDP	Markov Decision Process
OFDMA	Orthogonal Frequency-Division Multiple Access
PPO	Proximal Policy Optimization
RA-only	Resource-Allocation-only scheme
RSU	Roadside Unit
SAC	Soft Actor–Critic
SAGIN	Space–Air–Ground Integrated Network
V2R	Vehicle-to-RSU
VS-only	Vehicle-Selection-only scheme
w/o AoM	Without Age-of-Model weighting

## Appendix A. Proof of Lemma 1

Fix s, the other agents’ policies, the critic, the priority outputs, and the feasible discrete-action support. Let $q\left(a_{k}\right)={\bar{Q}}_{k}^{\mathrm{old}}\left(s,a_{k}\right)$ and $Z=\sum_{a_{k}}\exp\left(q\left(a_{k}\right)/\zeta_{k}\right)$, where the sum is over feasible discrete scheduling decisions. Assume 0<Z<∞. The normalized Boltzmann distribution is

(A1) $$p_{B}\left(a_{k}|s\right)=\frac{\exp(q\left(a_{k}\right)/\zeta_{k})}{Z}.$$

For any admissible local policy ($p\left(a_{k}\right)=\pi_{k}^{\prime }\left(a_{k}|s_{k}\right)$), expansion gives

(A2) $$\begin{array}{cc} D_{\mathrm{KL}}\left(p∥p_{B}\right)\; & =\sum_{a_{k}}p\left(a_{k}\right)\log p\left(a_{k}\right)-\frac{1}{\zeta_{k}}\sum_{a_{k}}p\left(a_{k}\right)q\left(a_{k}\right)+\log Z\\ \; & =-H\left(p\right)-\frac{1}{\zeta_{k}}E_{p}\left[q\left(a_{k}\right)\right]+\log Z. \end{array}$$

Since logZ is independent of the policy being optimized and $\zeta_{k}>0$, minimizing this KL divergence is equivalent to maximizing $E_{p}\left[q\left(a_{k}\right)\right]+\zeta_{k}H\left(p\right)$. The normalization is retained inside the KL distribution; only its additive contribution is omitted from the optimization. This proves the objective equivalence in Lemma 1. It does not prove a monotonic multi-agent policy-improvement bound.
